# Identification of European isolates of the lager yeast parent *Saccharomyces eubayanus*

**DOI:** 10.1093/femsyr/foac053

**Published:** 2022-12-07

**Authors:** Sean A Bergin, Stephen Allen, Conor Hession, Eoin Ó Cinnéide, Adam Ryan, Kevin P Byrne, Tadhg Ó Cróinín, Kenneth H Wolfe, Geraldine Butler

**Affiliations:** School of Biomolecular and Biomedical Science, Conway Institute, University College Dublin, Belfield, Dublin 4, Ireland; School of Biomolecular and Biomedical Science, Conway Institute, University College Dublin, Belfield, Dublin 4, Ireland; School of Biomolecular and Biomedical Science, Conway Institute, University College Dublin, Belfield, Dublin 4, Ireland; School of Medicine, Conway Institute, University College Dublin, Belfield, Dublin 4, Ireland; School of Biomolecular and Biomedical Science, Conway Institute, University College Dublin, Belfield, Dublin 4, Ireland; School of Medicine, Conway Institute, University College Dublin, Belfield, Dublin 4, Ireland; School of Biomolecular and Biomedical Science, Conway Institute, University College Dublin, Belfield, Dublin 4, Ireland; School of Medicine, Conway Institute, University College Dublin, Belfield, Dublin 4, Ireland; School of Biomolecular and Biomedical Science, Conway Institute, University College Dublin, Belfield, Dublin 4, Ireland

**Keywords:** European, lager parent, yeast, genome evolution

## Abstract

Lager brewing first occurred in Bavaria in the 15th century, associated with restrictions of brewing to colder months. The lager yeast, *Saccharomyces pastorianus*, is cold tolerant. It is a hybrid between *Saccharomyces cerevisiae* and *Saccharomyces eubayanus*, and has been found only in industrial settings. Natural isolates of *S. eubayanus* were first discovered in Patagonia 11 years ago. They have since been isolated from China, Tibet, New Zealand, and North America, but not from Europe. Here, we describe the first European strains UCD646 and UCD650, isolated from a wooded area on a university campus in Dublin, Ireland. We generated complete chromosome level assemblies of both genomes using long- and short-read sequencing. The UCD isolates belong to the Holarctic clade. Genome analysis shows that isolates similar to the Irish strains contributed to the *S. eubayanus* component of *S. pastorianus*, but isolates from Tibet made a larger contribution.

## Teaser

First isolation of European parents of lager yeasts from a wooded area on a university campus in Dublin, Ireland.

## Introduction

Brewing is one of the oldest industries associated with humans, with evidence of fermented beverages from China from 7000 to 8000 years ago, and from Israel from up to 13 000 years ago (Liu et al. 2018, 2019). Until the Middle Ages in Europe, most beer brewing was associated with *Saccharomyces cerevisiae* yeast, a ‘top fermenter’. The majority of ales today are brewed using *S. cerevisiae* (Gallone et al. 2016). Lagers first appeared in the 13th century, but became particularly common from the 15th century in Bavaria, where fermentation and storage took place in cool cellars (Gallone et al. 2019). The Beer Purity Laws in Bavaria in 1516 followed by the edict from Albrecht V in 1553 defined the components of beer and restricted brewing to the colder winter months, which may have selected for the ‘bottom fermenting’ cold tolerant lager yeasts now known as *Saccharomyces pastorianus* (Dornbusch 1998). Lager brewing spread worldwide in the 19th century, and lagers now represent > 90% of beers sold.

It has been known for some time that *S. pastorianus* is a hybrid of two parents, one of which is *S. cerevisiae* (de Barros Lopes et al. 2002, Dunn and Sherlock 2008). However, the second parent, *Saccharomyces eubayanus*, was not isolated until 2011, from the Patagonian Andes in South America (Libkind et al. 2011). *Saccharomyces eubayanus* and *S. pastorianus* isolates are cold tolerant (Gibson et al. 2013). Isolates of *S. eubayanus* have now been found in North America (Peris et al. 2014, Langdon et al. 2020), the Tibetan Himalayas, Sichuan and West China (Bing et al. 2014), and New Zealand (Gayevskiy and Goddard 2016). By far the most isolates have come from South America, where the species is associated with *Nothofagus* trees (Eizaguirre et al. 2018, Langdon et al. 2020, Nespolo et al. 2020). *Saccharomyces eubayanus* probably originated in Patagonia, and subsequently colonized the world (Langdon et al. 2020, Nespolo et al. 2020).

Phylogenetic analysis shows that *S. eubayanus* isolates fall into two main clades, PA and PB (Peris et al. 2016). PA is further divided into two lineages, PA-1 and PA-2, whereas PB can be divided into at least four lineages, PB-1, PB-2, PB-3, and the Holarctic clade (Peris et al. 2016, Langdon et al. 2020, Nespolo et al. 2020). Some PA/PB admixed isolates have also been identified (Peris et al. 2016, Langdon et al. 2020). Isolates from South America are very diverse and belong to all the main lineages, apart from the Holarctic clade. Isolates from outside South America belong to the PB-1 or Holarctic lineages, or are admixtures between PA and PB. Genome sequences from only a handful of isolates from the Holarctic lineage are available; two from Tibet (Bing et al. 2014, Brouwers et al. 2019a), and two from North Carolina, USA (Peris et al. 2016). However, the *S. eubayanus* component of the hybrid lager yeasts (*S. pastorianus*) also belongs to the Holarctic lineage, suggesting that the *S. eubayanus* parent originated in the Holarctic (defined as the nontropical parts of Europe and Asia, Africa north of the Sahara, and North America south to the Mexican desert region; Peris et al. 2016).

It is generally assumed that the original hybridization between *S. cerevisiae* and *S. eubayanus* occurred in a brewery, because no nonindustrial isolates of *S. pastorianus* have been discovered, and the *S. cerevisiae* parent is more similar to ale yeasts than to natural isolates (Gibson and Liti 2015, Monerawela et al. 2015, Krogerus et al. 2017, Gallone et al. 2019). It is, however, not clear how many hybridizations occurred. There are two main groups of *S. pastorianus* strains—Group I (Saaz) and Group II (Frohberg)—with differing fermentation properties (Gibson et al. 2013). Saaz strains are generally triploid, with approximately two genomes from *S. eubayanus* and one from *S. cerevisiae*, and Frohberg strains are tetraploid (or higher) with approximately equal contributions from *S. eubayanus* and *S. cerevisiae* (Walther et al. 2014). Lager yeast genomes have some chimeric chromosomes that contain junctions between DNA of *S. eubayanus* and *S. cerevisiae* origin, and some of these junctions are identical between Saaz and Frohberg strains, which strongly supports a single origin from a shared hybrid parent (Hewitt et al. 2014, Walther et al. 2014, Okuno et al. 2016, Gallone et al. 2019, Salazar et al. 2019). However, there is also evidence that some of the genetic variation in *S. eubayanus* populations survived through the bottleneck of hybridization and is retained in modern lager yeast genomes (Peris et al. 2016). This retention of standing genetic variation could be attributable to heterozygosity in the original hybridizing *S. eubayanus* parent, or to extra hybridizations or backcrossings that occurred after the original hybridization event shared by Saaz and Frohberg (Peris et al. 2016). Gallone et al. (2019) proposed that the initial hybridization event(s) occurred in the 1500s, around the time of the Bavarian Beer Purity Law, and that the Saaz and Frohberg lineages separated later. Both lineages went through a bottleneck following the purification of isolates by E.C. Hansen at the Carlsberg Laboratory in 1883 (Hansen 1883), and subsequent sharing between brewers (Gorter de Vries et al. 2019).

Regardless of when the hybridization(s) between *S. cerevisiae* and *S. eubayanus* occurred, they are likely to have occurred in Europe, and possibly in Bavaria. It is, therefore, surprising that no European isolates of *S. eubayanus* have been described. Climate modelling suggests that Europe is a prime location (Langdon et al. 2020). An intriguing metagenomics study from Alsammar et al. (2019) identified a small number of rDNA reads from *S. eubayanus* in the Italian Alps, suggesting that there might be a reservoir of the species there. Another indirect indication is that many European isolates of *Saccharomyces uvarum* associated with human-driven fermentation show evidence of introgression from *S. eubayanus* (Almeida et al. 2014). Here, we describe the discovery of the first European isolates of *S. eubayanus*. They were isolated from a university campus in Dublin, Ireland. They belong to the Holarctic lineage. As predicted by Peris et al. (2016), they match the lager yeasts at some, but not all, loci. We estimate that the *S. eubayanus* parent strains of the Saaz and Frohberg lineages shared approximately 54% of *S. eubayanus* alleles with a Tibet-like population, 40% with an Irish-like population, and 6% with a North American-like population. This supports the hypothesis that standing genetic variation in *S. eubayanus* persists in lager yeasts (Peris et al. 2016), and suggests that a pool of European isolates remains to be discovered.

## Methods

### Isolation of *S. eubayanus*

Detailed instructions are provided in Text S1 (Supporting Information). In brief, approximately 2 g of soil from two nearby locations on the UCD campus were inoculated in 10 ml yeast extract–peptone–dextrose (YPD) medium with chloramphenicol [30 μg/ml] and ampicillin [100 μg/ml] and incubated at room temperature for 1 week. A volume of 10 µl of culture was then used to inoculate 10 ml of fresh media, and incubated at room temperature for 38 h. Diluted culture was plated on YPD agar with chloramphenicol [30 μg/ml] and ampicillin [100 μg/ml] 4 days. Single colonies were streaked on agar plates, and tentatively identified by sequencing the ITS region. Two potential *S. eubayanus* isolates (UCD646 and UCD650) were chosen for genome sequencing.

### Genome sequencing

For short-read sequencing genomic DNA extraction, cells were harvested by centrifuging at 3000 rpm for 5 min and resuspending cell pellets in 200 µl Extraction buffer (2% Triton X 100, 100 mM NaCl, 10 mM Tris pH 7.4, 1 mM EDTA, and 1% SDS) in a screw-top tube. Nucleic acids were extracted by lysing cells and eluting DNA to an aqueous phase by adding ∼0.3 g acid washed beads and 200 µl phenol/chloroform/isoamyl alcohol 25:24:1, agitating the mixture with a 600 MiniG bead beater (Spex SamplePrep) for 30 s, and centrifuging at 14,000 rpm for 10 min. The top aqueous layer was extracted using 200 µl phenol/chloroform/isoamyl alcohol 25:24:1 and 200 µl TE buffer (10 mM TrisHCl, pH 8.0, 1 mM EDTA), and then extracted once more using 200 µl phenol/chloroform/isoamyl alcohol 25:24:1. A volume of 80 µl 7.5 M ammonium acetate and 1 ml 100% isopropanol were added to the total aqueous extraction to precipitate the DNA. DNA was pelleted by centrifugation at 14,000 rpm, washed using 70% ethanol and air-dried. Pellets were resuspended in 400 µl TE buffer with 1 µl RNase A (100 mg/ml) and incubated overnight at 37°C. DNA was reprecipitated and washed once more as above and resuspended in 150 µl water. Genomic DNA was sequenced by BGI Tech Solutions using DNBseq.

For long-read sequencing, the yeasts were cultured for 24–40 h in YPD medium. Genomic DNA was extracted using Qiagen’s Genomic Tip 100 G kit. High molecular weight was validated by gel electrophoresis, DNA concentration was quantified using a Qubit fluorometer, and quality was assessed using a Nanodrop. Multiplexed libraries using the SQK-RBK004 kit were made, and cleaned up with AMPure XP magnetic beads. Fresh MinION flow cells were primed with flow cell priming kit EXP-FLP002, and the libraries were sequenced using MinKNOW v4.1.22. Raw data was basecalled and demultiplexed after sequencing using Guppy v4.2.2.

### Genome assembly and variant calling

MinION read quality was assessed using Nanoplot, after which Nanofilt was used to retain only reads with Phred quality score (Q) and length (L) values of Q > = 10 and L > = 5 kb for UCD650, and Q > = 7 and L > = 1 kb for UCD646. Reads were then assembled using Canu version 2.2 (Koren et al. 2017), after which contigs were error-corrected by five rounds of NextPolish (Chen et al. 2021) using the DNBseq short reads. We discarded five small contigs (≤ 61 kb) from the UCD646 assembly, and two small contigs (≤ 37 kb) from the UCD650 assembly. These contigs were derived from rDNA, telomeric sequences, or the alternative allele at the MAT locus. The total length of the UCD646 assembly is 12 050 494 bp with an N50 of 914 339 bp and a GC content of 39.74%. The total length of the UCD650 assembly is 12 006 963 bp with an N50 of 916 217 and a GC content of 39.75%. Additional genome statistics are presented in Table S2 (Supporting Information).

Following annotation with YGAP (Proux-Wera et al. 2012), 5682 protein coding genes and 283 tRNAs were predicted in UCD646, and 5664 protein coding genes and 283 tRNAs were predicted in UCD650. Using BUSCO v5.1.2, genome completeness was estimated at 99.7% for both strains (compared to the Saccharomycetes lineage data set).

In addition to sequencing reads from *S. eubayanus* UCD646 and UCD650, WGS data for 87 additional *S. eubayanus* genomes, and for two *S. pastorianus* genomes were downloaded from SRA (Table S3, Supporting Information). Data from several strains sequenced by Langdon et al. (2020) were not included because the quality of the sequence was insufficient for accurate variant calling. Illumina and DNBseq reads were trimmed with Skewer version 0.2.2 using parameters ‘-m pe -t 4 -l 35 -q 30 -Q 30’ (Jiang et al. 2014). The trimmed reads were aligned using BWA MEM (version 0.7.23) to a FASTA file combining both the Canu-assembled UCD646 genome and the S288c_R64-3-1 *S. cerevisiae* reference genome. Read pairs that aligned to the *S. cerevisiae* sequence, and reads that had an identity < 90% to the region they mapped to, were removed. This was done to exclude reads from the *S. cerevisiae* fraction of the *S. pastorianus* genomes. The filtered BAM files were sorted and duplicate reads were marked using GenomeAnalysisToolkit (GATK version 4.0.1.2) SortSam and MarkDuplicates tools respectively (McKenna et al. 2010). Variants were called with GATK HaplotypeCaller using the tag ‘–genotyping_mode DISCOVERY’, combined using GATK CombineGVCFs and joint-genotyped using GATK GenotypeGVCFs. Variant files were filtered for read depth (< 15) and genotype quality (< 40) using GATK VariantFiltration. Additionally, clusters of SNPs (5 SNPs in a 20 bp window) were excluded using GATK VariantFiltration. GATK SelectVariants was used to exclude multiallelic sites, and sites where a genotype could not be determined for > 30% of strains. Finally, indels were excluded for tree construction using the ‘–select-type-to-include SNP’ tag.

### Phylogenetic analysis

A total of 1000 iterations of Random Repeated Haplotype Sampling (RRHS; Lischer et al. 2014) were used to generate SNP alignments where heterozygous sites were randomly resolved to either haplotype. SNP trees were then constructed from the 1000 alignment files using RAxML (v8.2.12) with the GTRGAMMA model of nucleotide substitution and the random number seed ‘-p 12345’ (Stamatakis 2014). The tree with the highest maximum likelihood was selected, and the remaining trees were used to generate branch support values.

### Identification of shared variants in *S. pastorianus* strains

A custom script (https://github.com/CMOTsean/Eubayanus_shared_variants) was used to identify variants present in *S. pastorianus* strains CBS 1538 and W34/70 that could be assigned to subpopulations of the Holarctic clade of *S. eubayanus*. Briefly, if a variant was found in *S. eubayanus* UCD646 and in *S. pastorianus* CBS 1538 or W34/70 but not in *S. eubayanus* Holarctic strains from Tibet (CDFM21L.1 and ABFM5L) or from the USA (yHRVM107 and yHRVM108) the strains were assumed to have uniquely shared ancestry at that variant. The analysis was then repeated using *S. eubayanus* CDFM21L.1 and yHRVM107 to assign ancestry to the Tibet and North Carolina lineages, respectively. For each of strains CBS 1538 and W34/70, blocks of shared ancestry were defined by two or more consecutive variants that were uniquely shared between the *S. pastorianus* strain and one of the Holarctic populations. The blocks were expanded outwards to cover the entire genome such that borders between blocks were drawn at the midpoint between outermost sites. The total length of each type of region was calculated to provide an estimate of the portions of the *S. eubayanus* component of the genome that have shared ancestry with each Holarctic population. The underlying data for each SNP is provided in summary in Table S1A (Supporting Information), with all data in Table S1B (Supporting Information).

### Growth on maltose

Three single colonies of *S. eubayanus* CBS 12357, UCD646, and UCD650 and *S. cerevisiae* S288C were inoculated in 5 ml of YPD and grown overnight with shaking at 200 rpm at 20°C. A total of two by 1 ml of each culture was centrifuged at 5000 rpm for 3 min, and cells were washed three times in 1 ml of PBS (phospho-buffered saline). One set of pellets was resuspended in 1 ml of YPD (2% glucose) and the other in 1 ml of YPM (2% maltose). The absorbance at 600 nm of each culture was adjusted to 0.1 and 200 µl was added to a round bottom 96-well plate. Cell growth was measured at room temperature every 10 min at 660 nm in a SYNERGY H1 microplate reader (BioTek Instruments, Inc.) The means of three biological replicates and the standard deviations are plotted. For spot assays, strains were grown overnight in 5 ml liquid YPD, washed three times in PBS and resuspended in 1 ml of PBS. The A_600_ was adjusted to 0.4 and then serially diluted by ⅕ seven times before spotting. Strains were grown at room temperature for 72 h.

## Results and discussion

**Figure 1.**
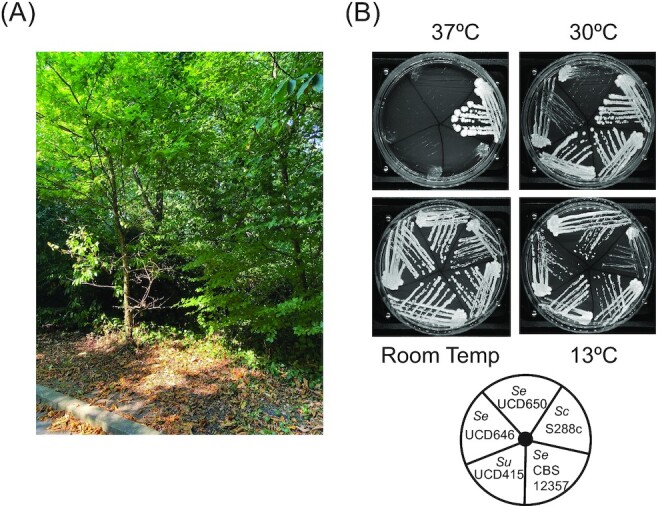
Identification and growth of *S. eubayanus* isolates. (A) *Saccharomyceseubayanus* UCD646 and UCD650 were identified in soil from a wooded area on the campus of University College Dublin. One soil sample was isolated from an area with oak, beech, and cherry laurel trees. (B) Isolates were streaked on YPD and incubated at the temperatures shown for 6 days. *Sc* = *S. cerevisiae; Su* = *S. uvarum* (UCD415 was also isolated from soil); and *Se* = *S. eubayanus*. CBS 12357 is the *S. eubayanus* type strain, obtained as VTT-C-72902. Room temperature is approximately 20°C.

Two *S. eubayanus* strains (UCD646 and UCD650) were isolated from soil samples from the Belfield campus of University College Dublin, as part of undergraduate research modules that identify and sequence the genomes of yeast species (Mullen et al. 2018, O'Boyle et al. 2018, Venkatesh et al. 2018, Almasoud et al. 2019, Faherty et al. 2019, Heneghan et al. 2019, O Cinneide et al. 2021, Ryan et al. 2021). A detailed description of the isolation method is provided in Text S1 (Supporting Information). The isolates came from soil on two sites on the university campus, about 17 m apart, collected in September 2021. One site (UCD650; GPS coordinates 53.306796, −6.233050) was soil under leaf litter from a sycamore tree (*Acer pseudoplatanus*), and the other site (UCD646; GPS coordinates 53.306563, −6.232683) was in soft soil close to oak (*Quercus robur*), beech (*Fagus sylvatica*), and cherry laurel (*Prunus laurocerasus*) trees (Fig. 1A). The isolates grow poorly at temperatures of 30°C or higher and grow well at cooler temperatures (e.g. 13°C), similar to *S. uvarum* and unlike *S. cerevisiae* (Fig. 1B). The type strain of *S. eubayanus* CBS 12357 grows better than either UCD646 or UCD650, particularly at 30°C (Fig. 1B).

The genomes of both isolates were sequenced using a combination of long-read (Oxford Nanopore MinION) and short-read (BGI DNBseq) sequencing. MinION reads were assembled using Canu (Koren et al. 2017), after which the assemblies were error-corrected by five rounds of polishing using NextPolish (Chen et al. 2021) with DNBseq reads. For both strains, each chromosome assembled as a single contig, except for chromosome XII where we manually made a join at the rDNA locus. Genomes were annotated using YGAP (Proux-Wera et al. 2012).

Both of the strains appear to be *MAT***a**/α diploids with very low heterozygosity. By mapping DNBseq reads to the final assemblies, we found eight heterozygous SNPs in UCD646, and 126 in UCD650. For UCD646, the *MAT* locus on chromosome III of the assembly has a *MAT***a** genotype, and the assembly also included a small contig with a *MAT*α allele. For UCD650, chromosome III contained a *MAT*α allele. There was no corresponding small contig with a *MAT***a** allele, but by BLASTN searches against a database of unassembled MinION reads we identified multiple reads that were derived from the *MAT* locus and were *MAT***a**. Chromosome III in both strains also contains *HML*α and *HMR***a** loci similar to the arrangement in *S. cerevisiae*.

Although the two strains were isolated from sites only a few metres apart, there are numerous small differences between the genomes of UCD646 and UCD650. They differ by 2517 homozygous SNPs. We identified 13 sites in the genome where one UCD strain contains a full-length Ty retrotransposon but the other does not (eight UCD646-specific Ty elements, and five UCD650-specific Ty elements). The strains also differ from each other by three inversions. On chromosome VII, UCD646 has an inversion of a 60-kb region spanning the centromere (all genes between *COG7/YGL005C* and *TIM21/YGR033C*) that is not present in UCD650, the Himalayan isolate CDFM21L.1 (Brouwers et al. 2019a), or the *S. eubayanus* type strain CBS 12357 from Patagonia (Baker et al. 2015, Brickwedde et al. 2018). The inversion is flanked by two Ty elements in opposite orientations in UCD646, only one of which is present in UCD650. On chromosome XI, UCD650 has an inversion of a 10-kb region (*YKT6/YKL196C* to *CNB1/YKL190W*) relative to UCD646, CDFM21L.1, and CBS 12357. On chromosome XIV, UCD650 differs from UCD646, CDFM21L.1, and CBS 12357 by a 25-kb inversion (*SIW14/YNL032W* to *ARK1/YNL020C*).

Both of the UCD strains contain the two reciprocal translocations that are shared by *S. eubayanus* and its sister species *S. uvarum* relative to *S. cerevisiae*: one translocation between chromosomes II and IV, and one between chromosomes VIII and XV (Scannell et al. 2011, Baker et al. 2015). Brouwers et al. (2019a) reported three major structural differences between the Himalayan isolate CDFM21L.1 and the type strain CBS 12357. We find that one of these (an 8-kb inversion in the chromosome VII-L subtelomere) is shared by the UCD strains and CDFM21L.1, and so differentiates the Holarctic from the Patagonian isolates. In the three Holarctic isolates, subtelomeres VI-R and VIII-L are highly similar to each other, whereas the type strain has a different sequence at VI-R, accounting for the second structural difference. The third structural difference noted by Brouwers et al. (2019a), involving an exchange between the chromosome V-R subtelomere and the rDNA region on chromosome XII, is specific to CDFM21L.1 and is not present in the UCD strains.

**Figure 2.**
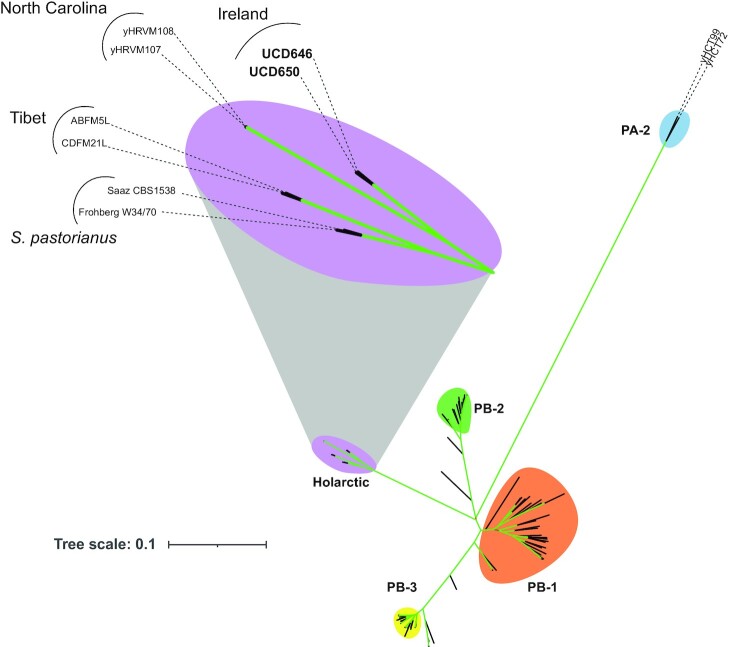
The Irish *S. eubayanus* isolates belong to the Holarctic clade. Unrooted maximum-likelihood tree of 89 *S. eubayanus* and two *S. pastorianus* strains was constructed with RAxML using the GTRGAMMA model of nucleotide substitution, using a concatenated SNP alignment of 319 298 SNP sites. Coloured shapes highlight distinct populations identified previously (Nespolo et al. 2020), and the two Irish (UCD) isolates are highlighted in bold. Most strain labels have been omitted for clarity (see Figure S1, Supporting Information). Branch colour denotes support values on a scale from 0 (red) to 100 (green). All branches in the Holarctic clade have a support value of 100. Support values < 100 are labelled in Figure 1 (Supporting Information).

To determine the relationship of the UCD strains to other *S. eubayanus* isolates, we constructed a phylogenetic tree using variants from the genomes of 89 other isolates, mostly from the PB clade (Fig. 2). This tree recapitulated previous phylogenetic trees (Peris et al. 2016, Langdon et al. 2020, Nespolo et al. 2020), in which the PB clade is divided into at least four main lineages, PB-1, PB-2, PB-3, and the Holarctic lineage. UCD646 and UCD650 are closely related and belong to the Holarctic lineage, together with two North Carolina isolates (yHRVM107 and yHRVM108; Peris et al. 2016), two isolates from Tibet (CDFM21L.1 and ABFM5L; Bing et al. 2014, Brouwers et al. 2019a), and the *S. eubayanus* components of the *S. pastorianus* lager yeasts CBS 1538 (Saaz) and W34/70 (Frohberg) (Peris et al. 2016).

**Figure 3.**
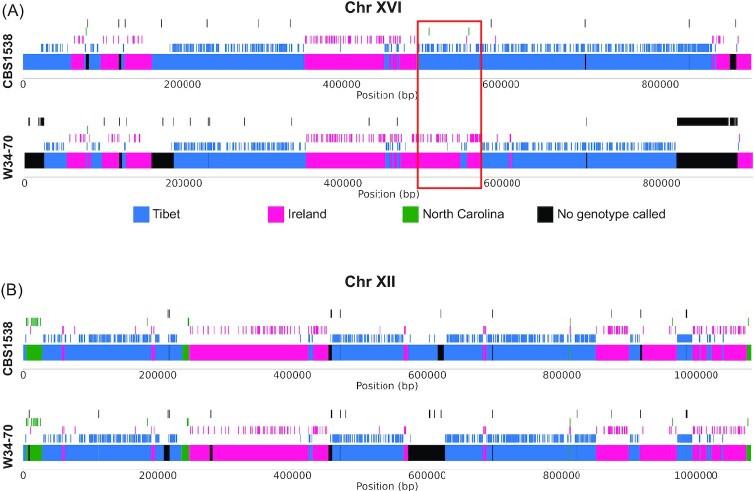
Shared ancestry of alleles between the *S. eubayanus* component of *S. pastorianus* genomes (CBS 1538 and W34/70) and Holarctic clade *S. eubayanus* isolates. Coloured offset vertical lines represent sites where an allele in a *S. pastorianus* strain is also present in only one other population (Tibet = Blue, Ireland = Magenta, or North Carolina = Green) in the Holarctic clade. Coloured rectangles represent blocks assigned to these populations, where blocks contain two or more neighboring alleles from the same population (borders are drawn at half the distance between the outermost sites). All alleles were called by alignment to the UCD646 assembly. Sites where a genotype could not be called or were filtered out are shown in black. All chromosome comparisons are shown in Figure S2 (Supporting Information), and supporting data is in Table S1 (Supporting Information). (A) Shared ancestry blocks of CBS 1538 and W34/70 on Chr XVI. CBS 1538 and W34/70 have similar patterns of ancestry except for a large region marked with a red box where CBS 1538 has shared ancestry with the Tibet population and W34/70 has shared ancestry with the Irish population. In addition, there are large regions in W34/70 where shared ancestry could not be reliably assigned. (B) Shared ancestry blocks of CBS 1538 and W34/70 on Chr XII. This example shows shared ancestry with all three Holarctic populations, although blocks assigned to North Carolina are shorter and fewer in number compared to Irish or Tibet blocks. Again, W34/70 has large blocks where no shared ancestry could be assigned.

The phylogenetic tree suggests that the Tibet isolates are more closely related to the lager yeasts than either the Irish or the North Carolina isolates, which cluster together (Fig. 2). However, recombination within or between populations can mask ancestry (Peris et al. 2016). We, therefore, compared the alleles along each chromosome in *S. pastorianus* CBS 1538 and W34/70 to the alleles from the Tibetan, North Carolina, and Irish isolates (Fig. 3; Figure S2 and Table S1, Supporting Information), to identify SNP sites at which a lager strain shares an allele with only one of the three Holarctic populations (see ‘Methods’). All sites were mapped onto the genome of UCD646. Figure 3 shows an example of allele conservation across two chromosomes, and all chromosomes are shown in Figure S2 (Supporting Information). Blocks of shared ancestry were defined by dividing each chromosome into runs of consecutive variants assigned to the same population. The total length of these blocks was used to estimate the proportion of the *S. eubayanus* component of CBS 1538 and W34/70 that has shared ancestry with each of the Holarctic populations. For CBS 1538, 4 188 309 bp (37.69%) were assigned to the Irish population, 6 233 324 bp (56.09%) were assigned to the Tibet population, and 690 505 bp (6.21%) were assigned to the North Carolina population. For W34/70, 4 525 481 bp (42.28%) were assigned to the Irish population, 5 558 609 bp (51.93%) were assigned to the Tibet population, and 619 169 bp (5.78%) were assigned to the North Carolina population. The underlying data for each SNP is provided in summary in Table S1A (Supporting Information), with all data in Table S1B (Supporting Information).

As reported by Peris et al. (2016), there are some regions where the Saaz isolate of *S. pastorianus* (CBS 1538) appears to have a different origin than the Frohberg isolate (W34/70). For example, one region of Chromosome XVI in CBS 1538 is more closely related to Tibetan isolates, whereas the equivalent region in W34/70 is more closely related to Irish isolates (boxed in Fig. 3). Other examples using the whole genome comparisons are shown in Figure S2 (Supporting Information).

The ability of *S. pastorianus* isolates to consume the sugars glucose, maltose, and maltotriose is important for lager fermentation (Zastrow et al. 2001). Maltose and maltotriose are transported by related proteins. In *S. pastorianus*, maltotriose transporters are encoded by *AGT1* and *MTY1; MTY1* is probably derived from recombination between ancestral maltose transporters (Baker and Hittinger 2019, Brouwers et al. 2019b). *Saccharomyces pastorianus* isolates have two *AGT1* alleles. One inherited from *S. cerevisiae* is truncated and nonfunctional, and the second full length allele is located on *S. eubayanus* chromosome XV (Vidgren et al. 2009). It was, therefore, assumed that *S. pastorianus AGT1* originated from *S. eubayanus*, even though *S. eubayanus* isolates cannot utilize maltotriose, and most lack an *AGT1* gene (Gibson et al. 2013, Baker and Hittinger 2019). Intact Agt1 maltotriose transporters were recently identified in Holarctic isolates of *S. eubayanus* from North America and from the Himalayas (Baker and Hittinger 2019, Brouwers et al. 2019a), and these are likely the source of the gene in *S. pastorianus*. There are no *AGT1* orthologs in the genomes of *S. eubayanus* UCD646 or UCD650.

In several *Saccharomyces* species, maltose utilization genes are organized in *MAL* loci, consisting of a maltose transporter (called *MALT* in *S. eubayanus*), a maltase enzyme (*MALS*) and a transcriptional regulator (*MALR*; Charron et al. 1989). The MalR regulator binds to a bidirectional promoter between *MALS* and *MALT* (Needleman 1991). The numbers of *MAL* loci, which are located in subtelomeric regions, vary between strains (Naumov et al. 1994).

**Figure 4.**
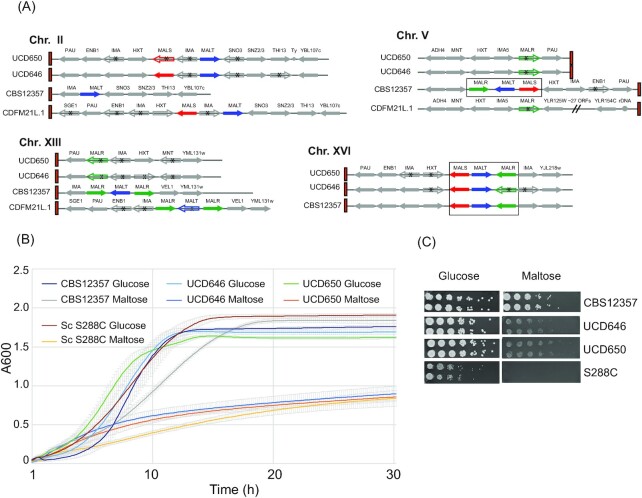
Maltose utilization in *S. eubayanus*. (A).Organization of maltose utilization genes. Telomeres are indicated with red boxes, and chromosomes with black lines. Maltose utilization genes are shown in colour, and other genes in grey. Intact *MAL* loci are highlighted with black boxes. Truncated open reading frames are shown as outlines with black asterisks. Ty = Ty element; rDNA = ribosomal DNA locus. Information for *S. eubayanus* CBS 12357 is from the assembly from Brickwedde et al. (2018) and for CDFM21L.1 is from Brouwers et al. (2019a). (B) Cultures of *S. eubayanus* CBS 12357, UCD646, and UCD650 and *S. cerevisiae* S288C were grown at room temperature in YP containing either 2% glucose or 2% maltose as the sole carbon source. Growth was monitored every 10 min. The mean and standard deviation of three biological replicates is shown. (C) Cells from the indicated strains were diluted ⅕ and spotted on YP agar with 2% glucose or 2% maltose and grown at room temperature for 72 h.

The *S. eubayanus* CBS 12357 type strain (isolated from Patagonia) encodes *MAL* transporters (*MALT*) at four subtelomeric regions (Brickwedde et al. 2018). Two (on Chrs V and XVI) are within intact *MAL* loci, with divergently transcribed *MALT* and *MALS* genes adjacent to *MALR* (Fig. 4A). *Saccharomyces eubayanus* CBS 12357 does not encode *AGT1*. The Tibetan (Himalayan) *S. eubayanus* isolates (e.g. CDFM21L.1, Fig. 4A) encode maltose utilization genes at three subtelomeric regions (Chrs II, V, and XIII) with shared synteny with CBS 12357, except that CDFM21L.1 has a rearrangement between Chr V and Chr XII, and has a fourth site on Chr XII (Brouwers et al. 2019a). There are *AGT1* genes at three other subtelomeres. None of the *MAL* loci in CDFM21L.1 are intact. *Saccharomyceseubayanus* UCD646 and UCD650 encode maltose utilization genes at four subtelomeric locations, that share synteny with both CDFM21L.1 and CBS 12357 (Fig. 4A). There is a *MAL* locus (*MALS, MALT*, and *MALR*) on Chr XVI (Fig. 4A). All the genes are intact in UCD650, but in UCD646 the *MALR* open reading frame is truncated because of a frameshift (Fig. 4A). UCD646 does not appear to encode a functional *MALR*; other copies on chromosomes V and XIII are also truncated (Fig. 4A). Several maltose utilization genes in *S. eubayanus* CBS 12357 also have stop codons/frameshifts (Brouwers et al. 2019a). We find that unlike the *S. eubayanus* CBS 12357 type strain, *S. eubayanus* UCD646 and UCD650 grow very poorly on media containing maltose as the sole carbon source (Fig. 4B and C). Growth on maltose is almost as poor as that of *S. cerevisiae* S288C, which has a defective *mal2* gene (Fig. 4B and C; Mortimer and Johnston 1986). It is, therefore, possible that the maltose transporters are not expressed in the Irish *S. eubayanus* isolates.

## Conclusion

Since the discovery of *S. eubayanus* isolates in Patagonia in 2011 (Libkind et al. 2011) it has been hypothesized that isolates would be found in Europe, and indeed modelling by Langdon et al. (2020) showed that Europe is a suitable location. Our discovery of isolates in Ireland is consistent with the ‘Out-of-Patagonia’ hypothesis, that *S. eubayanus* evolved in Patagonia where it adapted to cold and harsh conditions, and then spread to the rest of the world, probably in the postglacial period (Langdon et al. 2020, Nespolo et al. 2020). Our observation that some of the alleles in *S. pastorianus* isolates are closely related to alleles from the Irish *S. eubayanus* strains but that more of the genomes are close to alleles from Tibetan strains is consistent with the hypothesis that no one isolate of *S. eubayanus* is the direct ancestor of the parent of the lager yeasts, due to incomplete lineage sorting, backcrossing, or possibly multiple hybridization events (Peris et al. 2016). The maltotriose transporter gene *AGT1*, for example must have been acquired from isolates similar to the Tibetan strains. It is, therefore, likely that alleles in both Saaz and Frohberg strains of *S. pastorianus* are derived from standing variation in the *S. eubayanus* population (Peris et al. 2016).

However, it is also likely that isolates that share more similarities with *S. pastorianus* remain to be discovered in Europe. Langdon et al. (2020) speculate that competitive exclusion with *S. uvarum* or *Saccharomyces paradoxus* may have restricted the range of *S. eubayanus* in Europe, but it also possible that we are not looking in the right place, or the right ecological niche. This report, and the intriguing indication from metagenomics data from Italy (Alsammar et al. 2019), suggest that more European isolates from the Holarctic lineage will be discovered in the future.

## Supplementary Material

foac053_Supplemental_Files

## Data Availability

The data for this study have been deposited in the European Nucleotide Archive (ENA) at EMBL-EBI under BioProject accession number PRJEB55404. The annotated chromosome sequences have been deposited at DDBJ/ENA/GenBank with accession numbers OX291632-OX291648 for UCD646 and OX291491-OX291507 for UCD650. The raw reads were deposited at SRA with accession number ERR10084971 for UCD646 and ERR10084972 for UCD650.
