# Supplementary material for: Identification of European isolates of the lager yeast parent Saccharomyces eubayanus
Source: FEMS Yeast Res. 2022 Dec 7;22(1):foac053. doi: 10.1093/femsyr/foac053 (PMC9726447; doi:10.1093/femsyr/foac053)
Supplement: foac053_Supplemental_Files [file foac053_supplemental_files.zip › Suppl_Text1.docx]

**Supplementary Text 1. Isolation of *S. eubayanus***

The yeasts were isolated as part of teaching modules designed to introduce undergraduate students to research, and as such, they were not specifically designed to isolate *Saccharomyces eubayanus*. The modules were influenced by work from the Hittinger lab at the University of Wisconsin (https://hittinger.genetics.wisc.edu/Outreach/YEAST/). Most students participated in two modules. In the first (microbiology) module, students isolate yeast species from soil samples, and identify them by ITS sequencing. They then chose some isolates for whole genome sequencing (MinION sequencing carried out by PhD students in-house, and commercial DNB-seq, carried by BGI Genomics). In the second (bioinformatics) module, they assembled the genomes using both short- and long-read data.

The isolation methods chosen for the first module were restricted by the class schedule (Tuesday morning for 1 hour, Thursday morning for 3 hours, and Friday morning for 1 hour). The modules have been running for 4 years (with a break of one year for the Microbiology module due to Covid-19 restrictions). The isolation and inoculation methods have varied slightly from year to year. In 2021/2022, twelve students collected soil from the Belfield campus of University College Dublin. The campus was divided into zones, including tree-rich regions. Students collected ~10 soil samples each, at least one metre apart. They were directed to use a metal implement (spoon or trowel) to dig approximately 3 cm below the surface. Soil samples were stored in Nasco Whirl-pak 2 oz/58 ml write-on bags at room temperature, for 1-5 days. The implements were cleaned with sterile wipes between collections. The GPS coordinates of the collection sites were determined using Google Maps (or equivalent) on a mobile phone.

On Thursday of week 2, each soil sample was added to two 15 ml sterile Starstedt tubes (code 188261G), using enough soil to reach the line on the conical end of the tube (Fig. 1A). 9 ml of YPD containing chloramphenicol (30 μg/ml) and ampicillin (100 μg/ml) was added to each tube. The lids of the tubes were loosened slightly. One set of tubes was incubated at 30°C and one set was incubated at room temperature (approximately 20°C), both without shaking. The following Tuesday morning (after approximately 118 hours), 10 μl of each culture was transferred to 9 ml fresh YPD containing chloramphenicol (30 μg/ml) and ampicillin (100 μg/ml) and incubated at the same temperatures (30°C and room temperature) for approximately 48 h. On Thursday, each culture was diluted to 1/100 and 1/10,000 in sterile water, and 100 μl of each dilution was spread on YPD agar containing chloramphenicol (30 μg/ml) and ampicillin (100 μg/ml) and incubated at the original inoculation temperatures (30°C and room temperature) for 5 days, and stored at approximately 9°C. Over the next two weeks, potential yeasts (based on colony morphology, and on microscopic analysis for some) were streaked on YPD plates to obtain single colonies (Fig. 1B-D).


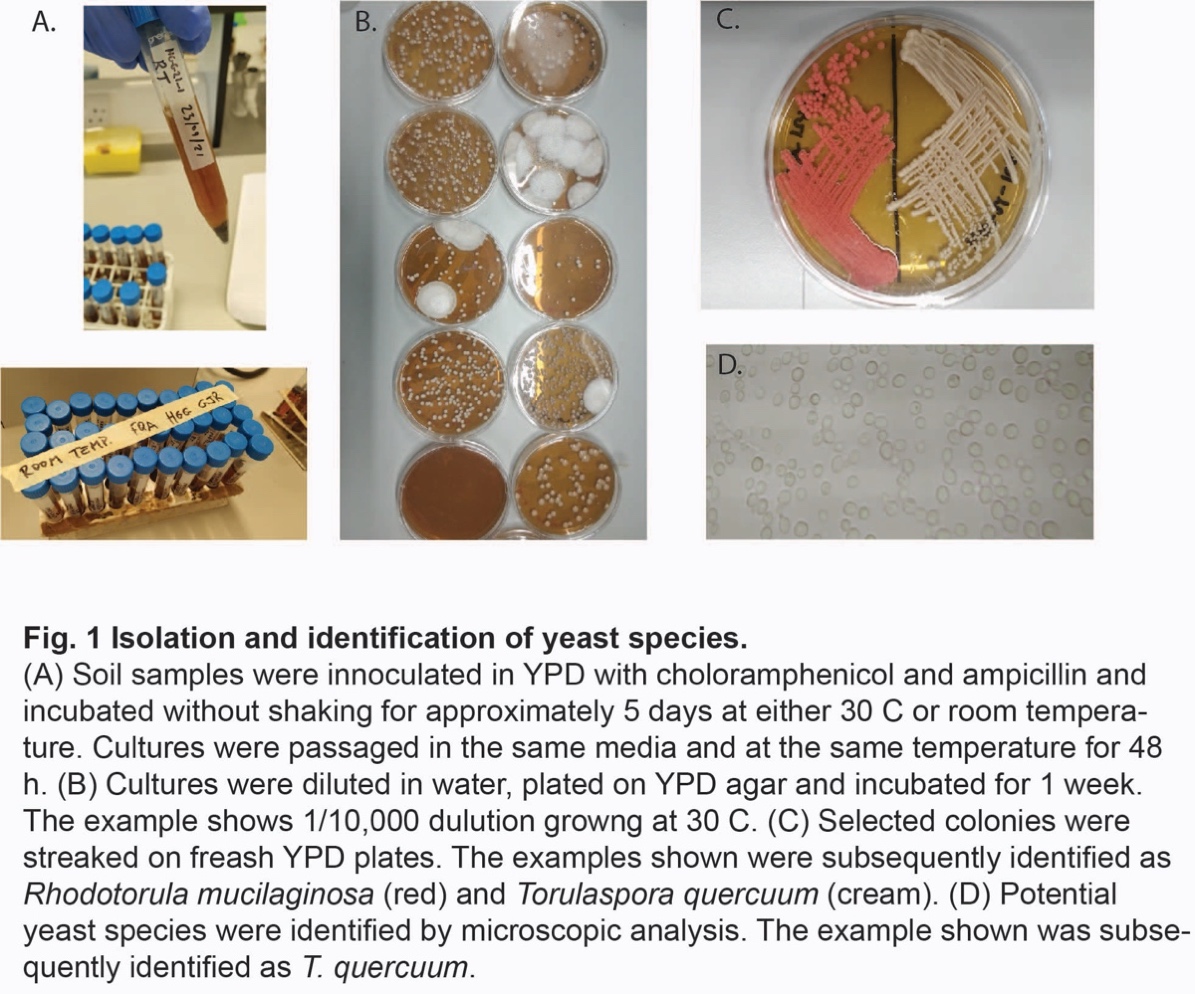


On Tuesday of week 6, all candidate isolates were patched on YPD plates and incubated at room temperature for 48 h. On Thursday, the ITS regions were amplified from the patched cultures in 25 µl reactions using MyTaq Red 2X from Bioline and primers ITS1 (5’ TCCGTAGGTGAACCTGCGG 3’) and ITS2 (5’ TCCTCCGCTTATTGATATGC 3’). Successful PCR products were identified by gel electrophoresis of 5 µl samples, the remainder was purified using NucleoSpin PCR purification kits, and sequenced from one end using ITS1 using Mix2Seq from Eurofins Genomics. In total, 94 samples from the 2021/22 class were successfully amplified, sequenced, and assigned a putative species identification based on >98% identity using BLAST at YeastIP (<http://genome.jouy.inra.fr/yeastip/>) and NCBI. Thirty-three different species were identified (Table 1); in some cases exact species identifications could not be assigned (e.g. *Candida oleophila* and *Candida railenensis* have similar ITS sequences). The most commonly isolated species were *Barnettozyma californica* (11 isolates) and *Kazachstania servazzii* (9 isolates); most other species were isolated only once (Table 1). For two isolates, UCD646 and UCD650, the closest ITS matches (>99% identity) were to *Saccharomyces bayanus* at YeastIP and *Saccharomyces pastorianus*, *Saccharomyces uvarum* X *Saccharomyces eubayanus* hybrid and *Saccharomyces bayanus* at NCBI (see sequences at end of document). Because the ITS sequences of *S. uvarum/S. bayanus/S. eubayanus* and *S. pastorianus* differ by only a few bases, part of the large subunit ribosomal RNA of isolate UCD646 was amplified using primers NL1 (5′-GCATATCAATAAGCGGAGGAAAAG-3′) and NL4 (5′-GGTCCGTGTTTCAAGACGG-3′). The closest matches at NCBI were *S. eubayanus*, *S. pastorianus*, and *Saccharomyces eubayanus* X *Saccharomyces uvarum* hybrids*.* UCD646 and UCD650 were then sequenced at the genome level, which confirmed that both were isolates of *S. eubayanus.*

Table 1 Identification of yeast species in 2021/22 soil samples.

| Species identification | No. isolates | Species identification | No. isolates |
| --- | --- | --- | --- |
| *Apiotrichum akiyoshidainum* | 2 | *Kazachstania servazzii* | 9 |
| *Apiotrichum dulcitum* | 5 | *Kluyveromyces dobzhanskii* | 2 |
| *Apiotrichum porosum* | 2 | *Kluyveromyces marxianus* | 1 |
| *Aureobasidium sp.* | 1 | *Lachancea thermotolerans* | 2 |
| *Barnettozyma californica* | 11 | *Metschnikowia zizyphicola* | 1 |
| *Blastobotrys aristata* | 1 | *Pichia kluyveri* | 1 |
| *Candida albicans* | 2 | *Rhodotorula mucilaginosa* | 3 |
| *Candida boidinii* | 5 | *Saccharomyces cerevisiae* | 2 |
| *Candida oleophila/railenensis* | 3 | *Saccharomyces eubayanus* | 2 |
| *Candida saitoana* | 2 | *Saccharomyces paradoxus* | 5 |
| *Cutaneotrichosporon moniliiforme* | 1 | *Saprochaete gigas* | 1 |
| *Cyberlindnera suaveolens* | 2 | *Teunomyces cretensis* | 1 |
| *Debaryomyces prosopidis* | 1 | *Torulaspora delbrueckii* | 5 |
| *Diutina catenulata* | 5 | *Torulaspora quercuum* | 6 |
| *Geotrichum candidum* | 1 | *Wickerhamomyces anomalus* | 3 |
| *Hanseniaspora uvarum* | 5 |  |  |
| *Kazachstania saulgeensis* | 1 |  |  |

ITS sequence *S. eubayanus* UCD646 (unedited)

ATTAAAATTTTGAATATTGGGATTTTTTTGTTTTGGCAAGAGCGTGAGAGCTTTTACTGGGCAAGAAGACAAGAGATGGAGAGTCCAGCCGGGCCTGCGCTTAAGTGCGCGGTCTTGCTAGGCTTGCAAGTTTCTTTCTTGCTATTCCAAACAGTGAGACTTCTCTGTTTTTGTTATAGGACAATTAAAACCGTTTCAATACAACACACTGTGGAGTTTTTATACTTTTGCAACTTTTTCTTTGGGTTTCGAGCAATCGAGCCCAGAGGTAACAAACACAAACAATTTTATTTATTCATTAAATTTTTGTCAAAAACAAGAATTTTCGTAACTGGAAATTTTAAAAATAATTAAAAACTTTCAACAACGGATCTCTTGGTTCTCGCATCGATGAAGAACGCAGCGAAATGCGATACGTAATGTGAATTGCAGAATTCCGTGAATCATCGAATCTTTGAACGCACATTGCGCCCCTTGGTATTCCAGGGGGCATGCCTGTTTGAGCGTCATTTCCTTCTCAAACATTCTGTTTGGTAGTGAGTGATACTCTCTGGAGTTAACTTGAAATTGCTGGCCTTTTCATTGGATGTTTTTTTTCCAAAGAGAGGTTTCTCTGCGTGCTTGAGGTATAATGCAAGTACGGTCGTTTTAGGTTTTACCAACTGCGGCTAATCTTTTTTGTACTGAGCGTATTGAAACGTTATCGATAAGAAGAGAGCGTCTAGGCGAACAATGTTCTTAAAGTTTGACCTCAAATCAGGTAGGAGTACCCGCTGAACTTAAGCATATCATAAAGCC

ITS sequence *S. eubayanus* UCD650 (unedited)

AAATAAGAATTAAAGTTTTGATATTGGATTTTTTTGTTTTGGCAAGAGCGTGAGAGCTTTTACTGGGCAAGAAGACAAGAGATGGAGAGTCCAGCCGGGCCTGCGCTTAAGTGCGCGGTCTTGCTAGGCTTGCAAGTTTCTTTCTTGCTATTCCAAACAGTGAGACTTCTCTGTTTTTGTTATAGGACAATTAAAACCGTTTCAATACAACACACTGTGGAGTTTTTATACTTTTGCAACTTTTTCTTTGGGTTTCGAGCAATCGAGCCCAGAGGTAACAAACACAAACAATTTTATTTATTCATTAAATTTTTGTCAAAAACAAGAATTTTCGTAACTGGAAATTTTAAAAATAATTAAAAACTTTCAACAACGGATCTCTTGGTTCTCGCATCGATGAAGAACGCAGCGAAATGCGATACGTAATGTGAATTGCAGAATTCCGTGAATCATCGAATCTTTGAACGCACATTGCGCCCCTTGGTATTCCAGGGGGCATGCCTGTTTGAGCGTCATTTCCTTCTCAAACATTCTGTTTGGTAGTGAGTGATACTCTCTGGAGTTAACTTGAAATTGCTGGCCTTTTCATTGGATGTTTTTTTTCCAAAGAGAGGTTTCTCTGCGTGCTTGAGGTATAATGCAAGTACGGTCGTTTTAGGTTTTACCAACTGCGGCTAATCTTTTTTGTACTGAGCGTATTGAAACGTTATCGATAAGAAGAGAGCGTCTAGGCGAACAATGTTCTTAAAGTTTGACCTCAAATCAGGTAGGAGTACCCGCTGAACTTAAGCATATCATAAAGCGGAAGGAAAAGATTC
