# Supplementary material for: Identification of European isolates of the lager yeast parent Saccharomyces eubayanus
Source: FEMS Yeast Res. 2022 Dec 7;22(1):foac053. doi: 10.1093/femsyr/foac053 (PMC9726447; doi:10.1093/femsyr/foac053)
Supplement: foac053_Supplemental_Files [file foac053_supplemental_files.zip › SuppTable3.docx]

Supplementary Table 3: Sequences used in this study

| **Strain** | **Species** | **Accession** |
| --- | --- | --- |
| CBS1538 | *S. pastorianus* | SRR18163228 |
| W34-70 | *S. pastorianus* | SRR18163229 |
| CL1001.1 | *S. eubayanus* | SRR9003304 |
| CL1002.1 | *S. eubayanus* | SRR9003281 |
| CL1003.1 | *S. eubayanus* | SRR9003260 |
| CL1004.1 | *S. eubayanus* | SRR9003288 |
| CL1005.1 | *S. eubayanus* | SRR9003251 |
| CL1006.1 | *S. eubayanus* | SRR9003299 |
| CL1007.1 | *S. eubayanus* | SRR9003244 |
| CL1008.1 | *S. eubayanus* | SRR9003301 |
| CL1009.1 | *S. eubayanus* | SRR9003302 |
| CL1010.1 | *S. eubayanus* | SRR9003238 |
| CL1101.1 | *S. eubayanus* | SRR9003277 |
| CL1104.1 | *S. eubayanus* | SRR9003294 |
| CL1106.1 | *S. eubayanus* | SRR9003242 |
| CL1108.1 | *S. eubayanus* | SRR9003259 |
| CL1109.1 | *S. eubayanus* | SRR9003298 |
| CL1110.1 | *S. eubayanus* | SRR9003256 |
| CL1111.1 | *S. eubayanus* | SRR9003262 |
| CL1112.1 | *S. eubayanus* | SRR9003306 |
| CL204.3 | *S. eubayanus* | SRR9003273 |
| CL210.1 | *S. eubayanus* | SRR9003291 |
| CL211.3 | *S. eubayanus* | SRR9003292 |
| CL212.2 | *S. eubayanus* | SRR9003307 |
| CL215.1 | *S. eubayanus* | SRR9003289 |
| CL216.1 | *S. eubayanus* | SRR9003261 |
| CL218.1 | *S. eubayanus* | SRR9003271 |
| CL220.3 | *S. eubayanus* | SRR9003313 |
| CL221.1 | *S. eubayanus* | SRR9003312 |
| CL248.1 | *S. eubayanus* | SRR9003235 |
| CL444.2 | *S. eubayanus* | SRR9003305 |
| CL446.2 | *S. eubayanus* | SRR9003269 |
| CL447.3 | *S. eubayanus* | SRR9003265 |
| CL449.1 | *S. eubayanus* | SRR9003266 |
| CL450.1 | *S. eubayanus* | SRR9003245 |
| CL451.4 | *S. eubayanus* | SRR9003268 |
| CL467.2 | *S. eubayanus* | SRR9003237 |
| CL471.2 | *S. eubayanus* | SRR9003279 |
| CL601.1 | *S. eubayanus* | SRR9003233 |
| CL604.1 | *S. eubayanus* | SRR9003286 |
| CL605.1 | *S. eubayanus* | SRR9003300 |
| CL606.1 | *S. eubayanus* | SRR9003303 |
| CL607.1 | *S. eubayanus* | SRR9003290 |
| CL608.1 | *S. eubayanus* | SRR9003255 |
| CL610.1 | *S. eubayanus* | SRR9003248 |
| CL611.1 | *S. eubayanus* | SRR9003309 |
| CL619.1 | *S. eubayanus* | SRR9003249 |
| CL620.1 | *S. eubayanus* | SRR9003258 |
| CL621.1 | *S. eubayanus* | SRR9003264 |
| CL701.1 | *S. eubayanus* | SRR9003254 |
| CL702.1 | *S. eubayanus* | SRR9003284 |
| CL703.2 | *S. eubayanus* | SRR9003236 |
| CL704.2 | *S. eubayanus* | SRR9003287 |
| CL705.1 | *S. eubayanus* | SRR9003253 |
| CL706.2 | *S. eubayanus* | SRR9003241 |
| CL710.1 | *S. eubayanus* | SRR9003232 |
| CL711.2 | *S. eubayanus* | SRR9003285 |
| CL715.1 | *S. eubayanus* | SRR9003247 |
| CL801.1 | *S. eubayanus* | SRR9003250 |
| CL803.1 | *S. eubayanus* | SRR9003295 |
| CL804.1 | *S. eubayanus* | SRR9003283 |
| CL807.1 | *S. eubayanus* | SRR9003240 |
| CL810.1 | *S. eubayanus* | SRR9003239 |
| CL812.1 | *S. eubayanus* | SRR9003280 |
| CL813.1 | *S. eubayanus* | SRR9003274 |
| CL814.1 | *S. eubayanus* | SRR9003275 |
| CL815.1 | *S. eubayanus* | SRR9003252 |
| CL816.1 | *S. eubayanus* | SRR9003267 |
| CL817.1 | *S. eubayanus* | SRR9003278 |
| CL821.1 | *S. eubayanus* | SRR9003297 |
| CL824.1 | *S. eubayanus* | SRR9003246 |
| CL835.1 | *S. eubayanus* | SRR9003263 |
| CL902.1 | *S. eubayanus* | SRR9003314 |
| CL903.1 | *S. eubayanus* | SRR9003310 |
| CL904.1 | *S. eubayanus* | SRR9003272 |
| CL905.1 | *S. eubayanus* | SRR9003296 |
| CL906.1 | *S. eubayanus* | SRR9003276 |
| CL907.1 | *S. eubayanus* | SRR9003311 |
| CL909.1 | *S. eubayanus* | SRR9003234 |
| CL910.1 | *S. eubayanus* | SRR9003293 |
| CL915.1 | *S. eubayanus* | SRR9003308 |
| CL916.1 | *S. eubayanus* | SRR9003257 |
| CRUB1568T | *S. eubayanus* | SRR1217601 |
| CDFM21L | *S. eubayanus* | SRR10208683 |
| ABFM5L | *S. eubayanus* | SRR10208684 |
| yHCT99 | *S. eubayanus* | SRR3294507 |
| yHRVM107 | *S. eubayanus* | SRR3294512 |
| yHRVM108 | *S. eubayanus* | SRR2586159 |
| yHCT72 | *S. eubayanus* | SRR2586152 |
| UCD646 | *S. eubayanus* | TBA |
| UCD650 | *S. eubayanus* | TBA |
