# Supplementary material for: Identification of European isolates of the lager yeast parent Saccharomyces eubayanus
Source: FEMS Yeast Res. 2022 Dec 7;22(1):foac053. doi: 10.1093/femsyr/foac053 (PMC9726447; doi:10.1093/femsyr/foac053)
Supplement: foac053_Supplemental_Files [file foac053_supplemental_files.zip › SuppFig1.pdf]

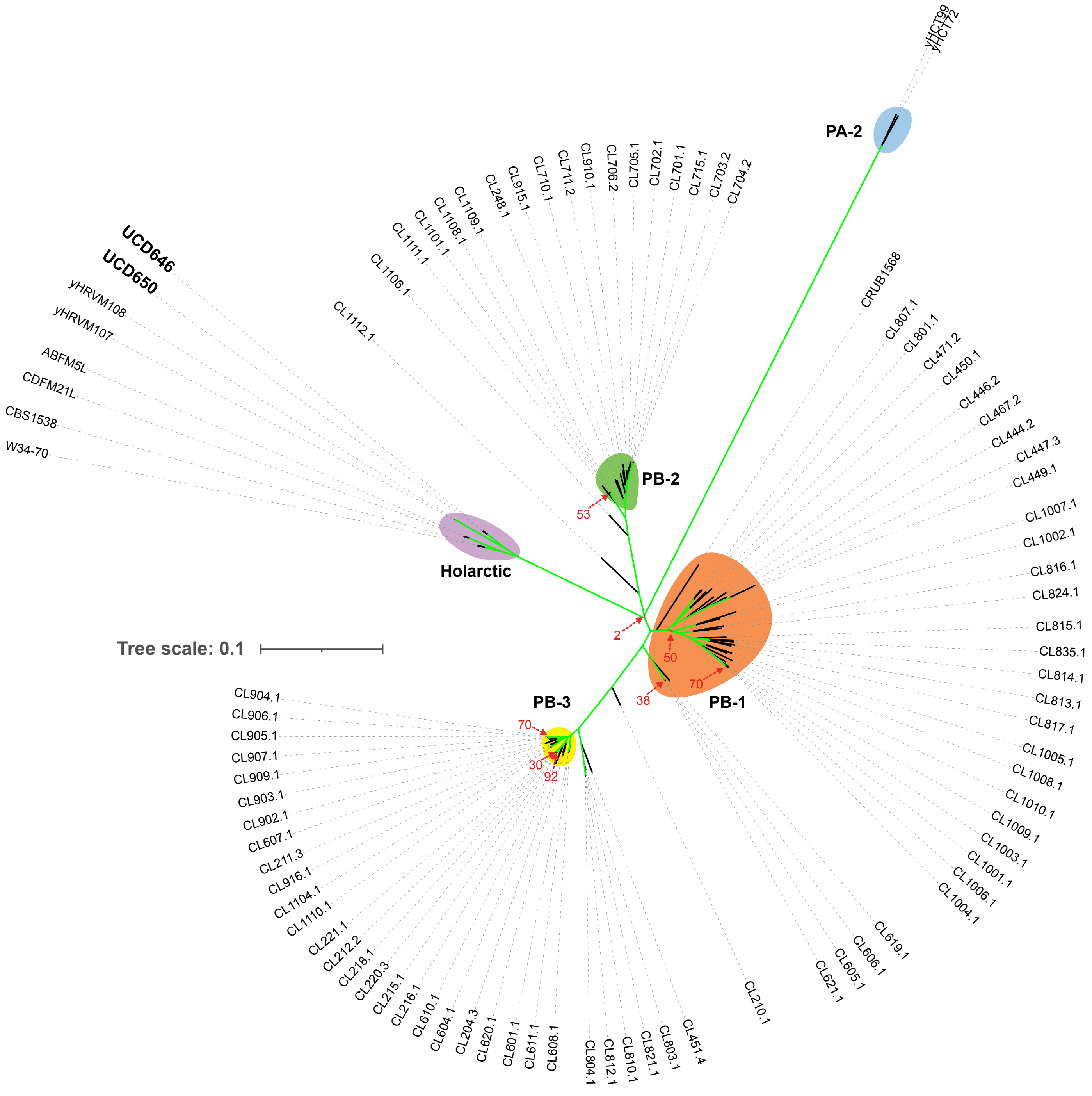

Supplementary Fig. 1. Estimated phylogeny of 89 *S. eubayanus* and two *S. pastorianus* strains. The tree was constructed with RAxML using the GTRGAMMA model of nucleotide substitution, using a concatenated SNP alignment. Coloured shapes highlight distinct populations identified previously (Nespolo et al. 2020), and the two UCD isolates are highlighted in bold. All branches in the Holarctic clade have a support value of 100. Support values < 100 are labelled with red arrows.
