# Supplementary material for: Identification of European isolates of the lager yeast parent Saccharomyces eubayanus
Source: FEMS Yeast Res. 2022 Dec 7;22(1):foac053. doi: 10.1093/femsyr/foac053 (PMC9726447; doi:10.1093/femsyr/foac053)
Supplement: foac053_Supplemental_Files [file foac053_supplemental_files.zip › SuppFig2.pdf]

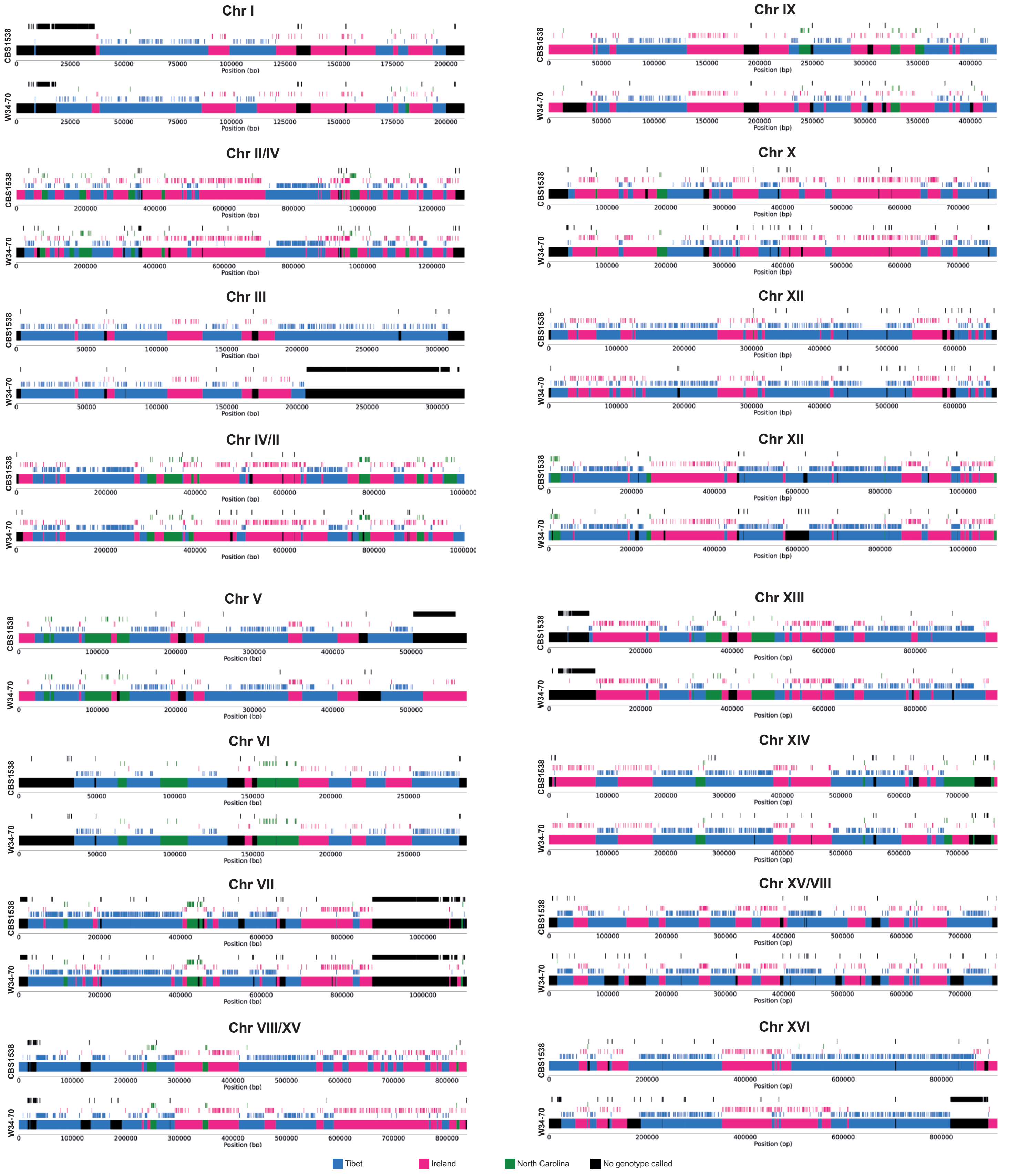

Supplementary Figure 2.

Shared ancestry of alleles between the *S. eubayanus* component of *S. pastorianus* genomes (CBS 1538 and W34/70) and Holarctic clade *S. eubayanus* isolates. Coloured offset vertical lines represent sites where an allele in a *S. pastorianus* strain is also present in only one other population (Tibet-=Blue, Ireland=Magenta, or North Carolina=Green) in the Holarctic clade. Coloured rectangles represent blocks assigned to these populations, where blocks contain two or more neighboring alleles from the same population (borders are drawn at half the distance between the outermost sites). All alleles were called by alignment to the UCD646 assembly. Sites where a genotype could not be called or were filtered out are shown in black. Supporting data is provided in Supplementary Table 1.
